# Posterior Dislocation of the Shoulder: The Light-Bulb Sign

**DOI:** 10.7759/cureus.47800

**Published:** 2023-10-27

**Authors:** Christos Koutserimpas, Maria Piagkou, Ilias Karaiskos, Efstathios Chronopoulos, Nikolaos-Achilleas Arkoudis

**Affiliations:** 1 Orthopaedics and Traumatology, 251 Hellenic Air Force General Hospital, Athens, GRC; 2 Anatomy/Oral Surgery, National and Kapodistrian University of Athens, Athens, GRC; 3 Orthopaedic Surgery, National and Kapodistrian University of Athens, Athens, GRC; 4 Orthopaedic Surgery, Konstantopoulio General Hospital, Nea Ionia, GRC; 5 Radiology, Research Unit of Radiology and Medical Imaging, Medical School, National and Kapodistrian University of Athens, Athens, GRC

**Keywords:** shoulder arthroplasty, hill sachs lesion, shoulder dislocation, shoulder anatomy, shoulder instability

## Abstract

Posterior dislocation is a rather rare injury, often misdiagnosed. The current report offers valuable insights regarding the anatomical background of this clinical entity and emphasizes the 'light-bulb sign,' which can be observed in anterior-posterior shoulder X-rays when there is a posterior dislocation. It is crucial for healthcare professionals, including emergency department physicians, radiologists, general practitioners, orthopedic surgeons, and other relevant medical experts, to be well-acquainted with this sign and maintain a heightened awareness when encountering such cases. A 57-year-old male presented to the Emergency Department due to right shoulder pain immediately after an epileptic seizure. His arm was locked in internal rotation, while the initial X-rays, although did not reveal evident malalignment, showed the light-bulb sign. Further imaging with a computer tomography (CT) scan exhibited a large (50%) reverse Hill-Sachs defect. The patient was treated surgically with hemiarthroplasty. The light-bulb sign should be a red flag for physicians who evaluate these patients or these X-rays. The patient’s history, such as epileptic seizures and examination, especially the locked arm in internal rotation, are of paramount importance for not misdiagnosing these cases.

## Introduction

The first posterior shoulder dislocation was reported in 1741 by White et al., while, later on, Mclaughlin described the clinical presentation of this clinical entity, which ranges from recurrent episodes of subluxation to locked dislocations [1,2]. Posterior shoulder instability is much less frequent than the anterior one [1,3].

Regarding the anatomical background, the shoulder joint is often characterized as the least congruent joint in the human body, often likened to a golf ball resting on a tee [4]. In reality, only around one-third of the humeral head makes contact with the glenoid at any given moment. This limited bony constraint allows the shoulder to have an extensive range of motion for everyday tasks [4,5]. Consequently, the stability of the shoulder hinges on a dynamic interplay between static, including the glenoid labrum, the articular congruity, the glenohumeral ligaments, the joint capsule, and the negative intra-articular pressure, as well as the dynamic stabilizing structures, including the rotator cuff muscles [4,5].

Posterior dislocation typically occurs as a result of high-energy shoulder trauma, which may include incidents such as seizures, electrocution, or forces applied from the rear [1].

The present report highlights the light-bulb sign that may be evident in the anterior-posterior shoulder X-rays in cases of posterior shoulder dislocation. In particular, the light-bulb sign may appear in cases where the humerus dislocates posteriorly and it also internally rotates such that the head contour projects like a light-bulb when depicted in the anterior-posterior X-ray views. Physicians at the emergency departments, general practitioners, orthopaedic surgeons, and radiologists should be familiar with this sign and have a high level of awareness in cases with an indicated medical history. Furthermore, the anatomical background of the posterior dislocation of the shoulder is discussed.

## Case presentation

A 57-year-old male presented to the Emergency Department due to right shoulder pain immediately after an epileptic seizure (first episode). The patient upon presentation was hemodynamically stable (blood pressure: 136/78 mmHg, heart rate: 90 beats/min, respiratory rate: 12 breaths/min), afebrile (36.8 °C), and alert. His right arm was locked in a slightly internally rotated position, while external rotation could not be passively or actively performed. There were no neurovascular deficits. The absence of normal glenohumeral joint contour was also observed.

**Figure 1:**
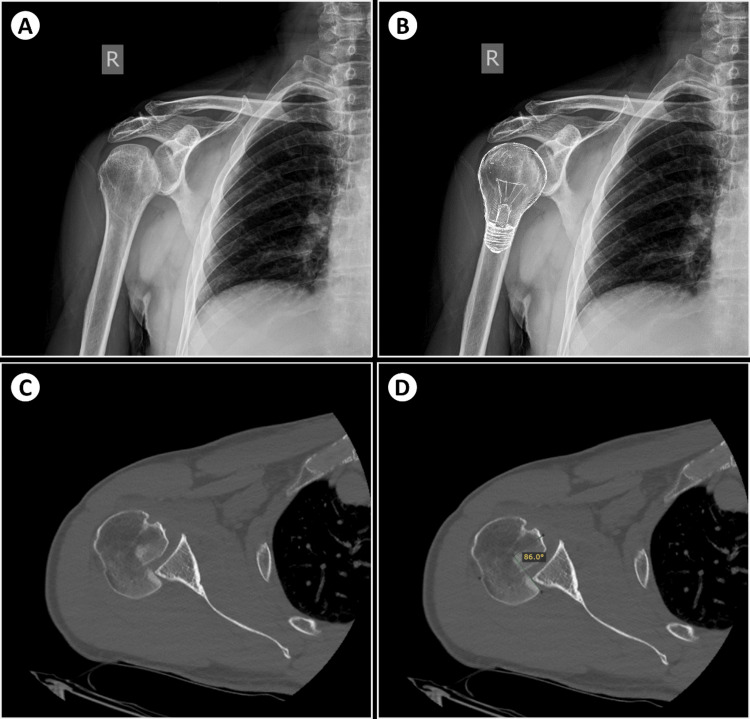
X-ray and computer tomography revealing the light-bulb sign and the Hill-Sachs defect, respectively. Figure 1 (A). Anteroposterior x-ray of the right shoulder, although not clearly evident of glenohumeral joint misalignment, displays the humeral head assuming a light bulb-like configuration, thus indicating the possibility of posterior shoulder dislocation. The parallelism is more easily perceived in illustration (B), which exhibits a light bulb drawing layered over the posteriorly dislocated humeral head. (C) An axial image of a computer tomography (CT) scan of the right shoulder joint confirmed the diagnosis of posterior shoulder dislocation and additionally demonstrated a synchronous large reverse Hill-Sachs lesion. (D) Measurements show that approximately 50% of the articular surface of the humeral head is affected by the reverse Hill-Sachs fracture lesion (almost 90°), as per the Cicak et al. method [6].

The initial X-ray view, although it did not clearly depict a glenohumeral joint misalignment, demonstrated the “light-bulb sign” (the head of the humerus in the same axis as the shaft producing a lightbulb shape, due to internal rotation) (Figure 1A), which is indicative of posterior shoulder dislocation (as illustrated in Figure 1B). Further investigation with a computer tomography (CT) scan confirmed the posterior shoulder dislocation (Figure 1C), as well as a concurrent large reverse Hill-Sachs lesion (impaction fracture of the anteromedial aspect of the humeral head), affecting 50% of the articular surface (Figure 1D). The patient was treated with closed reduction under sedation at the Emergency Department.

**Figure 2:**
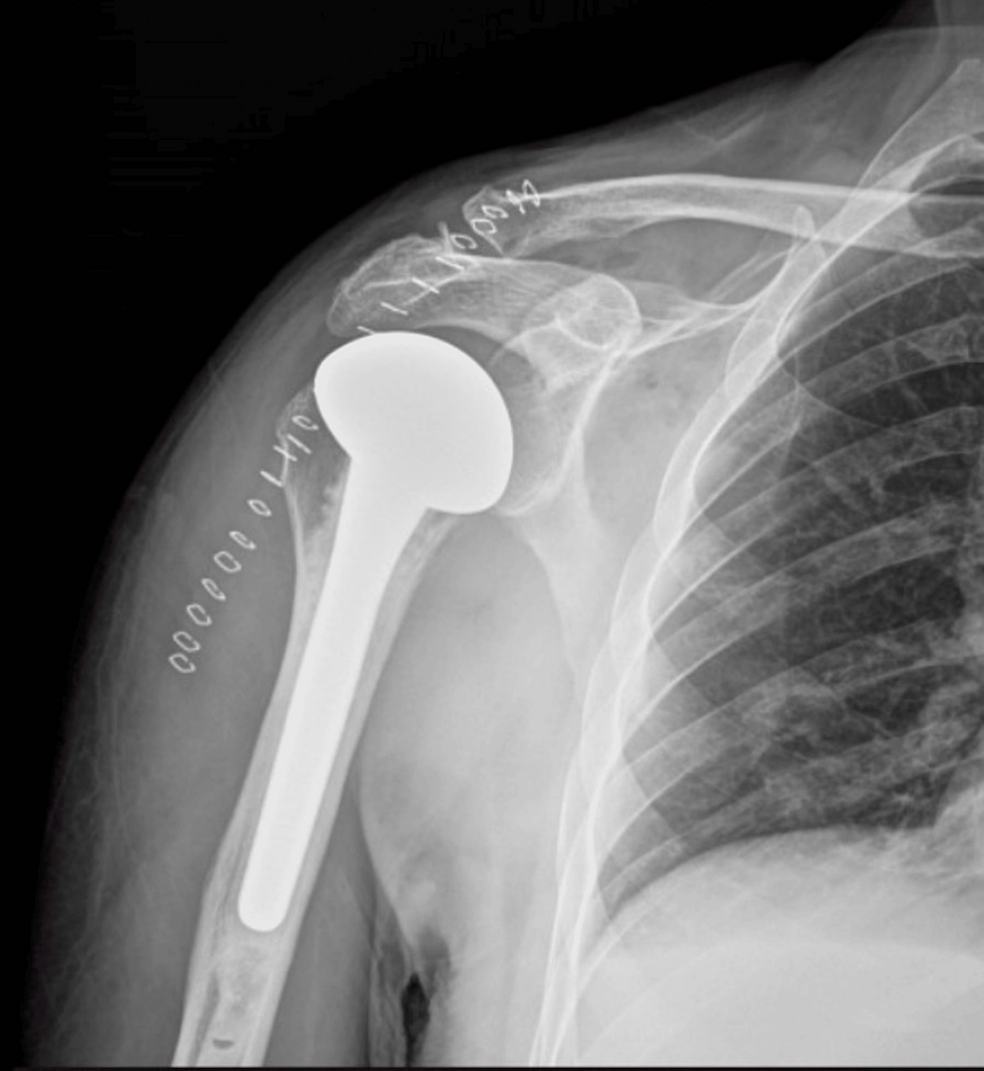
Postoperative anteroposterior x-ray of the right shoulder. The patient has been treated with hemiarthroplasty.

Due to persistent instability (subluxation of the shoulder in passive abduction and internal rotation) and the large humeral head deficit, surgical treatment was proposed. Four days after initial admission, the shoulder surgery was performed. Under general anesthesia, the patient was placed in a beach chair position. Under antimicrobial prophylaxis (1.5 g cefuroxime), a deltopectoral approach was performed. Osteotomy of the humeral head followed and cemented hemiarthroplasty was performed (FH Arrow™). Figure 2 shows the final result.

The patient made an uneventful recovery and was discharged on the second postoperative day.

## Discussion

The shoulder girdle serves as an intricate connection point linking the upper extremity to the central skeleton. Among all the significant joint dislocations encountered in emergency departments, the glenohumeral joint of the shoulder stands out as the most frequent, accounting for roughly half of all cases [1,7]. Posterior shoulder dislocations make up approximately 2%-5% of all shoulder dislocations. Among those, recurrent posterior dislocations are observed in around 30% of patients, increasing the likelihood of degenerative changes in the joint [8].

Regarding the anatomical background and pathophysiology of this clinical entity, the shoulder girdle houses the glenohumeral joint, characterized as a ball-and-socket joint [4,5]. This joint is unique due to its shallow nature, with the glenoid fossa of the scapula being roughly one-third the size of the humeral head. The shoulder ranks as the body's most flexible joint, boasting an extensive range of motion. However, this shallowness, which enables such remarkable mobility, also renders the joint more susceptible to instability [4,9]. To enhance stability, the cartilaginous labrum encircles the glenoid's rim, deepening the socket. Additionally, the joint capsule, ligaments, and muscular attachments play crucial roles in maintaining stability. Notably, the nerves of the brachial plexus and subclavian vessels traverse the anterior shoulder girdle, situated between the first rib and clavicle, potentially making them vulnerable to injury [4,5,9]. Nonetheless, such injuries are less common in posterior dislocations. Dislocations can arise from various shoulder injuries, including trauma, blunt force, or twisting motions. The most frequent mechanism of posterior shoulder dislocation involves forceful adduction with internal rotation and flexion, although a direct, blunt impact to the anterior shoulder can also result in dislocation [10].

The posterior dislocation of the shoulder represents an easily misdiagnosed clinical entity, usually affecting highly demanding individuals (30-55 years old), resulting from forced muscle contraction, as in epileptic seizures, electric shock or electroconvulsive therapy, major trauma such as motor vehicle accidents, or other injuries involving axial loading of the arm, in an adducted, flexed, and internally rotated position [8,10]. It should be noted that the large internal rotators, such as the latissimus dorsi and pectoralis major, are stronger than the external rotators, such as the infraspinatus and teres minor, which could explain the association between epileptic seizures and posterior shoulder dislocation. Patients with posterior shoulder dislocation present with limited external rotation in cases of acute posterior dislocation, while the shoulder may be locked in an internally rotated position, especially in undiagnosed dislocations [4,5,9].

Posterior dislocations can be categorized into three anatomical types based on where the humeral head ultimately comes to rest: (1) subacromial, which is the most common; (2) subglenoid; and (3) subspinous. Typically, in posterior dislocations, the humeral head ends up positioned posteriorly relative to the glenoid and inferiorly in relation to the acromion [1,7,11,12]. Posterior shoulder dislocations can lead to complications such as osteonecrosis, posttraumatic arthritis, and restricted joint mobility. Osteonecrosis of the humeral head can manifest following a straightforward dislocation, but it is more frequently observed in cases involving fracture dislocations of the anatomic neck [7,13]. The reverse Hill-Sachs lesion is associated with locked and difficult-to-reduce dislocations, while the tuberosity fracture and the avulsion of the posterior band of the inferior glenohumeral ligament with acute posterior dislocation or subluxation are lesser [5,10,11,14]. Furthermore, the reverse Bankart lesion is associated with the posterior shoulder dislocation, and it is characterized by detachment of the posterior-inferior capsulolabral complex, while posterior glenoid rim fracture and posterior labral cysts have been linked to the chronic reverse Bankart lesion [8,10].

Initial diagnosis is missed in emergency departments in up to 79% of cases, due to the lack, in most times, of a traumatic event, as well as the not-so-evident malalignment in the X-ray view [7]. Increased suspicion of this clinical entity is required especially in emergency departments, since delayed diagnosis and management may have deleterious effects on the shoulder function. The scapular “Y” view may also be performed to indicate the diagnosis, while CT scans could also be considered when suspected posterior dislocations are not seen on radiographs [1,10]. Besides confirming the diagnosis, CT scans can better delineate the affected structures and are, therefore, especially valuable for assessing the extension of the reverse Hill-Sachs humeral head defect, which is important for the accurate classification and, thus, treatment of such cases [15].

In order to estimate the extent of the reverse Hill-Sachs lesion, in our case, measurements were performed on the CT scan obtained employing the method of Cicak et al. [6]. The angle between the posterior edge of the defect and the line connecting the two edges of the articular surface of the humeral head was measured. By interpreting this method, a small defect of <25% of the humeral head articular surface would have an angle of less than 45 degrees; a medium defect of between 25% and 50% of the humeral head articular surface would have an angle of more than 45 degrees but less than 90 degrees; and a large defect of more than 50% of the humeral head articular surface would require an angle of more than 90 degrees.

Magnetic resonance imaging (MRI) may provide improved visualization of labral, ligamentous, and joint capsule injuries due to its enhanced ability to accurately depict soft tissues [1,8].

Regarding treatment of posterior dislocation of the shoulder, acute reduction and immobilization in 10-20 degrees of external rotation for four weeks should be attempted for the majority of acute cases, followed by physical therapy, including rotator cuff strengthening and periscapular stabilization [8,10]. Surgical treatment may also be considered in some cases. In recurrent cases of young patients, despite the appropriate course of physical therapy, arthroscopic posterior labral repair may be performed [1,16]. In cases of excessive glenoid retroversion, posterior glenoid wedge osteotomy is indicated, while, in chronic cases with reverse Hill-Sachs defect < 40%, open reduction with subscapularis transfer (McLaughlin) or lesser tuberosity transfer to the defect (modified McLaughlin) is indicated [1,2,13,17]. In cases of reverse Hill-Sachs defect > 40% and in chronic cases, hemiarthroplasty may be performed, while, in such cases that suffer also glenoid arthritis, total shoulder arthroplasty could be proposed. In the present patient, due to the large reverse Hill-Sachs defect (50%), hemiarthroplasty was the treatment of choice [8,10].

## Conclusions

Posterior dislocation of the shoulder is a rather uncommon injury, while the lack of a traumatic event leads to misdiagnosis in almost half of all cases. Furthermore, the malalignment in the initial X-ray view may not be evident. The present report highlighted the light-bulb sign that should be a red flag for physicians who evaluate these patients or these X-rays. The patient’s history, such as epileptic seizures and examination, especially the locked arm in internal rotation, is of utmost importance for not misdiagnosing these cases.
